# *Senecio ovatus* poisoning in a horse – A case report

**DOI:** 10.17221/37/2024-VETMED

**Published:** 2024-09-22

**Authors:** Andrea Kopecka, Tereza Novotna, Zdenka Svobodova, Zuzana Drabkova

**Affiliations:** ^1^Equine Clinic, Faculty of Veterinary Medicine, University of Veterinary Sciences Brno, Brno, Czech Republic; ^2^Department of Animal Protection and Welfare and Veterinary Public Health, Faculty of Veterinary Hygiene and Ecology, University of Veterinary Sciences Brno, Brno, Czech Republic

**Keywords:** equine, histopathology, chromatography, intoxication, pyrrolizidine alkaloid, wood ragwort

## Abstract

This study describes a case of poisoning by pyrrolizidine alkaloids in a horse. To the best of the author’s knowledge, this is the first confirmed case of *Senecio ovatus* poisoning. A six-year-old 450-kg Irish cob mare was presented to the Equine Clinic of the University of Veterinary Sciences Brno (Czechia) with symptoms of hepatic encephalopathy, which progressively worsened with time despite intensive therapy and led to euthanasia. A complex diagnostic and therapeutic approach including the post-mortem patoanatomical and histopathological examination is described here. Regarding the histopathology of the liver, there was necrosis with haemorrhage, fatty changes and inflammation. A later inspection of the grazing area revealed the presence of *Senecio ovatus* (wood ragwort). A sensitive chromatographic method was used to determine the pyrrolizidine alkaloids and their metabolites in the plasma and the liver. In both of the samples, metabolites of pyrrolizidine alkaloids were detected. Although pyrrolizidine alkaloid poisoning was proven, the histopathological findings typical for this disease were absent. It is clear from our case that the histopathology in cases of poisoning by pyrrolizidine alkaloids may not always be conclusive.

Horses are among the most susceptible species to poisoning by *Senecio* spp. and other plants containing pyrrolizidine alkaloids (PAs) (Kalac and Kaltner 2021). Poisoning occurs when animals mistake the early rosettes for adjacent forage, when other forage is absent or when hay is contaminated with dried plant parts, which are more palatable and less easily avoided (Pohlmann et al. 2005; Cortinovis and Caloni 2015). PAs require liver metabolisation to exert toxicity and, for this reason, this organ is the most affected (Field et al. 2015; Geburek et al. 2020). Lungs and kidneys have also been identified as target organs of PA metabolites in humans and animals (Moreira et al. 2018). Poisoning, which can manifest itself after weeks or months after ingestion, is characterised by hepatic insufficiency, secondary photosensitisation and central nervous system (CNS) derangement due to the elevated blood ammonia (Panter et al. 2007; Cortinovis and Caloni 2015; Anadon et al. 2018). A liver biopsy has the characteristic findings of megalocytosis, centrilobular necrosis, portal fibrosis and biliary hyperplasia (Divers 2014). Diagnosis of *Senecio* spp. toxicosis in horses may be difficult due to the insidious onset of the clinical signs and because the toxic plants may no longer be present in the pasture or feed by the time the clinical signs become evident (Pohlmann et al. 2005). The aim of this report is to describe a clinical case of fatal poisoning of a horse with fresh *Senecio ovatus* plants. Although the histopathological findings were not typical, poisoning by PAs was proven by the chromatographic determination of their metabolites.

## Case description

### HISTORY

**Figure 1:**
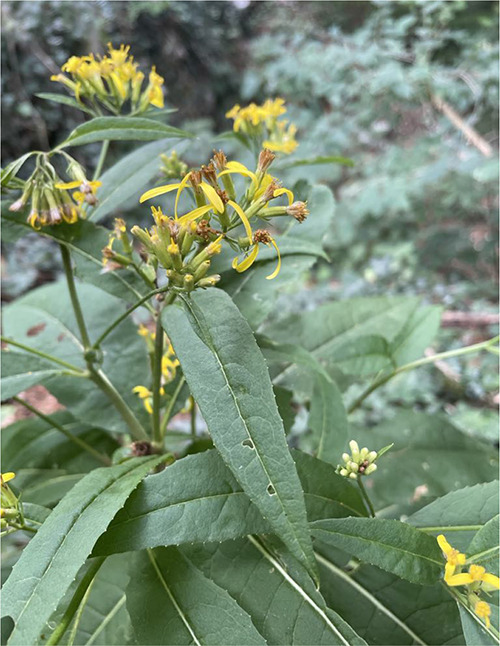
*Senecio ovatus* plants in the forest accessible from the pasture

A six-year-old Irish cob mare weighing 450 kg was found on the pasture, dyspnoeic and reluctant to move. She was last seen by the owners three days before when she was completely normal. Two weeks before that, she had been vaccinated against tetanus and influenza. The whole herd, consisting of 28 horses of the same breed, was kept on the pasture; no additional feed was given. Some other horses were showing signs of photosensitivity, one horse was presented with severely elevated alkaline phosphatase (14 μkat/l, ref. range 2.45–4.35 μkat/l), gamma glutamyl transferase (3 μkat/l, ref. range 0.02–0.67 μkat/l) and aspartate amino transferase (34 μkat/l, ref. range 1.7–5.83 μkat/l). Six months prior to the onset of the above- mentioned clinical signs, the herd was released into a small forest connected to the pasture for the first time. A later examination of the forest revealed multiple *Senecio ovatus* (wood ragwort) plants (Figure 1).

### CLINICAL FINDINGS

The mare was apathetic, with an elevated rectal temperature (39.9 °C) and icteric mucous membranes. The rectal examination revealed a distended urinary bladder; a large volume of yellow urine was collected after catheterisation. Dexamethasone (Colvasone; Norbrook laboratories limited, Ireland; 0.05 mg/kg i.v.) was administered by the field veterinarian, and the patient was referred to the Equine Clinic of the University of Veterinary Sciences Brno, Czechia. The mare was brought to the clinic in lateral recumbency, tachypnoeic (56 breaths/min), tachycardic (120 beats/min), with elevated rectal temperature (40 °C). The conjunctivae were hyperaemic and the oral mucosae pale to icteric. The capillary refill time was prolonged and not measurable. The mare was presented with signs of acute respiratory distress.

### BLOOD ANALYSES

A jugular venipuncture was performed to obtain a blood sample for the acid-base status, haematology and biochemistry. There was severe metabolic acidosis, hyperlactataemia, hyperchloraemia, hypocalcaemia and hyperglycaemia. The acid-base status was evaluated again one hour later and then six hours after admission, but no significant improvement was seen (Table 1).

**Table 1:** Analysis of the acid-base status

Parameters	Values at admission	Values 6 h after admission	Reference ranges
pH	** 7.186↓**	** 7.198↓**	7.360–7.430
cK^+^ (mmol/l)	3.8	** 3.4↓**	3.5–4.6
cNa^+^ (mmol/l)	140	** 149↑**	136–142
cCa^2+^ (mmol/l)	** 1.05↓**	** 1.15↓**	1.40–1.70
cCl^–^ (mmol/l)	** 108↑**	** 110↑**	98–104
cGlu (mmol/l)	** 15.6↑**	** 3.4↓**	4.1–6.4
cLactate (mmol/l)	** 11.8↑**	** 9.5↑**	1.0–2.0
Alactic base excess, c (mmol/l)	**–14.7↓**	**–10.1↓**	–2.0–4.0
Anion Gap, c (mmol/l)	** 19.2↑**	** 19.8↑**	1.5–11.5

The haematology showed leukocytosis, neutrophilia, monocytosis, lymphocytosis, elevated packed cell volume and haemoglobin. All the values remained almost unchanged six hours after admission (Table 2).

**Table 2:** Haematological analysis

Parameters	Values at admission	Values 6 h after admission	Reference ranges
Total erythrocytes (× 10^12^/l)	9.56	9.47	6.2–10.2
Packed cell volume (l/l)	** 0.501↑**	** 0.464↑**	0.31–0.43
Haemoglobin (g/l)	** 169↑**	** 173↑**	111–159
Total leukocytes (× 10^9^/l)	** 27.9↑**	** 31.31↑**	6.0–10.0
Neutrophils (× 10^9^/l)	** 21.1↑**	** 25.67↑**	3.4–5.4
Lymphocytes (× 10^9^/l)	** 4.610↑**	** 3.76↑**	2.0–3.2
Monocytes (× 10^9^/l)	** 2.030↑**	** 1.88↑**	0.2–0.4
Eosinophils (× 10^9^/l)	0.030	0	0–0.4
Platelets (× 10^9^/l)	192	240	100–250

The biochemistry revealed increased concentrations of the total protein and a lower albumin, an elevated total bilirubin and the increased activity of gamma glutamyl transferase, aspartate amino transferase, lactate dehydrogenase and creatine kinase.

When re-examined six hours later, all the values remained outside the reference ranges and, in addition, a venous blood sample for the ammonia determination was transported immediately to the laboratory. The concentration of ammonia was markedly increased. The bile acids and alkaline phosphatase were determined, which were also elevated (Table 3).

**Table 3:** Biochemical analysis

Parameters	Values at admission	Values 6 h after the admission	Reference ranges
Total protein (g/l)	**95.3↑**	**77↑**	53–73
Albumin (g/l)	** 28.6↓↓**	** 21.7↓**	29–41
Aspartate amino transferase (μkat/l)	** 76.56↑**	** 64.91↑**	1.7–5.83
Creatine kinase (μkat/l)	** 26.58↑**	** 60.89↑**	1.83–4.17
Lactate dehydrogenase (μkat/l)	** 33.39↑**	** 47.61↑**	3.75–11.67
Gamma glutamyl transferase (μkat/l)	** 7.17↑**	** 5.5↑**	0.02–0.67
Alkaline phosphatase (μkat/l)	not analysed	** 14.52↑**	2.45–4.35
Urea (mmol/l)	2.6	4.3	2.5–10.0
Creatinine (μmol/l)	92	** 183.2↑**	85–165
Total bilirubin (μmol/l)	** 113.6↑**	** 164.1↑**	13–34
Bile acids (μmol/l)	not analysed	** 87.2↑**	1–8.5
Ammonia (μmol/l)	not analysed	** 325.96↑**	< 60

### URINALYSIS

Five litres of urine were obtained by catheterisation. It was straw-yellow, viscous, with a pH of 6 and a specific gravity of 1.024. Blood and leucocytes were present at low levels (+), haemoglobin was negative.

### ENDOSCOPIC AND ULTRASONOGRAPHIC EXAMINATION

Bilateral laryngeal paralysis was confirmed endoscopically. Due to the persistent lateral recumbency, transthoracic and transabdominal ultrasonographic exams were limited. Minor comet-tail artefacts were observed in an accessible area of the lung field, otherwise, no significant findings were seen.

## Treatment

On admission in the trailer, an emergency tracheostomy with a subsequent intratracheal administration of 100% oxygen was started and gradually decreased. Hypertonic saline (prepared by pharmacy Sv. Anna, Brno, Czech Republic; 1.1 ml/kg i.v.) was given followed by sodium bicarbonate (8.4% sodium bicarbonate; B. Braun Melsungen AG, Germany; 1.7 ml/kg i.v), calcium gluconate 20% (prepared by pharmacy Sv. Anna, Brno, Czech Republic; 1.1 ml/kg i.v.) and isotonic saline (natrium chloride Baxter 0.9% infusion solution; Baxter Czech spol. s.r.o., Prague, Czech Republic; 22 ml/kg i.v.), all administered separately. Diazepam (Apaurin; KRKA d.d., Ljubljana, Slovenia; 0.02 mg/kg i.v.) was given for seizure control at the trailer. For transportation to the stall, a combination of 2% xylazine (Xylazin Ecuphar; Bioveta a.s., Ivanovice na Hané, Czech Republic; 1.1 mg/kg i.v.), diazepam (Apaurin; KRKA d.d., Ljubljana, Slovenia; 0.06 mg/kg i.v.) and 10% ketamine (Narkamon; Bioveta a.s., Ivanovice na Hané, Czech Republic; 2.2 mg/kg i.v.) was used. Flunixin meglumine (Flunbix; Biopharm a.s., Jílové by Prague, Czech Reppublic; 1.1 mg/kg i.v.) and furosemide (Furosemid BBP; BB Pharma a.s., Prague, Czech Republic; 1 mg/kg i.v.) were administered later.

### OUTCOME

The mare showed no clinical improvement over the course of six hours and the blood parameters remained practically unchanged. Euthanasia was recommended to the owner and subsequently performed.

## Post-mortem findings

**Figure 2:**
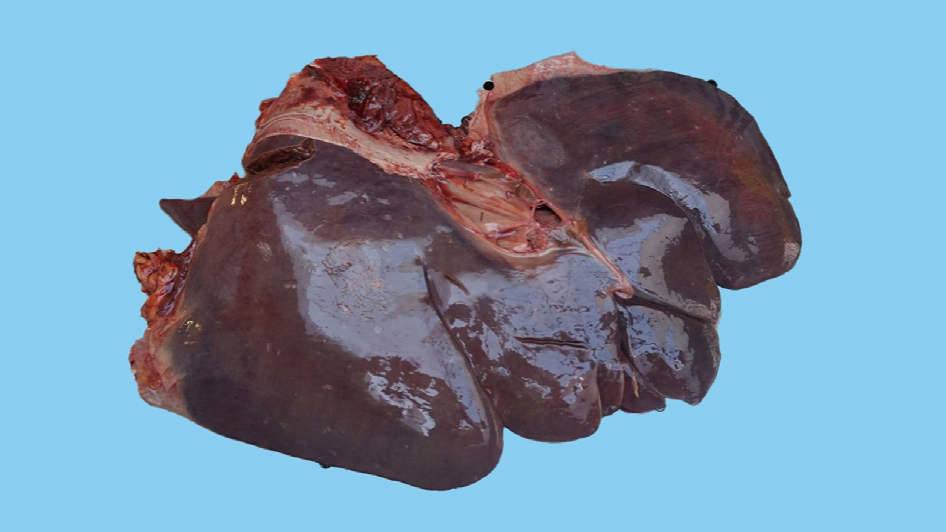
Gross appearance of the liver with macroscopic focal changes to the liver parenchyma

An autopsy with a subsequent histopathological examination was performed within 24 h after the euthanasia at the Department of Pathological Morphology and Parasitology of the University of Veterinary Sciences Brno, Czechia. Macroscopically, white focal changes of the liver parenchyma were present (Figure 2).

The histology revealed a partial autolysis of the hepatocytes of various intensities in different areas, sinusoidal dilatation and focal marked hyperaemia of the parenchyma. Multifocal vacuolar dystrophy with intracytoplasmatic droplets was apparent in the non-autolysed hepatocytes. In some parts of the interstitium, smaller foci of a chronic round cell inflammatory reaction (lymphocytes, plasmatic cells, macrophages) and rarely some granulocytes were present.

**Figure 3:**
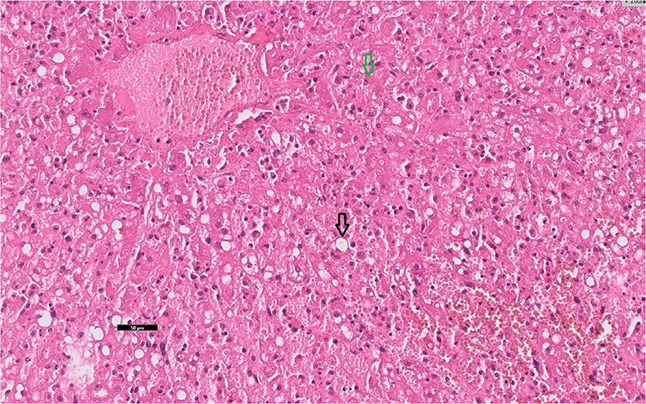
Histopathological appearance of the liver parenchyma Vacuolation (black arrow) and necrotic hepatocyte (green arrow) (FINN Pathologists, Norfolk, United Kingdom)

Samples were sent to FINN Pathologists, Norfolk, United Kingdom, for a second opinion analysis, which revealed marked and panlobular acute to subacute hepatic necrosis with haemorrhage and fatty changes with some very slight sparing of the periportal hepatocytes in some regions (Figure 3). Accompanying the necrosis, there was extensive congestion and a small number of admixed leucocytes (neutrophils and a few macrophages).

### CHROMATOGRAPHIC ANALYSIS

**Figure 4:**
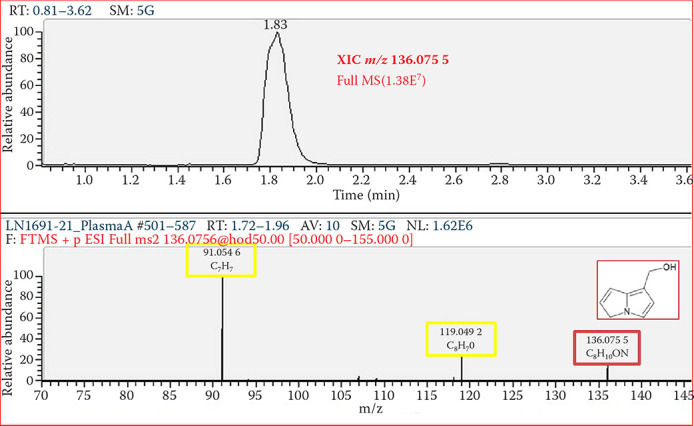
Extracted ion chromatogram (XIC) of ion m/z 136.075 5 and its fragmentation spectrum including the structural formula

The blood plasma collected immediately prior to euthanasia and a liver sample were frozen at –80 °C and sent for analysis of the PAs and their metabolites to the Department of Food Analysis and Nutrition, University of Chemistry and Technology, Prague, Czechia. Ultraefficient liquid chromatography with a high-resolution tandem mass spectrometry technique (UHPLC-HRMS/MS) was chosen. A non-targeted analysis was carried out for screening of potential PA metabolites, based on previously published protocols (Ruan et al. 2015; Geburek et al. 2020; Zhu et al. 2022). In both the plasma and the liver samples, the PAs were not quantified, but a relatively intense signal corresponding to the elemental composition of the PA metabolites (m/z 136.075 5, C_8_H_10_ON) described in a previous study (Geburek et al. 2020) was recorded (Figure 4) and several other potential PA metabolites were detected. The paper describing the identification strategy of the PA metabolites in detail is being prepared for publishing (Brabenec et al. 2024).

### DIAGNOSIS

A suspected diagnosis of *S.* *ovatus* poisoning was initially made based on the history, clinical presentation and presence of the plant in the nearby forest. The diagnosis was confirmed by UHPLC-HRMS/MS determination of the PA metabolites in the liver and plasma.

## DISCUSSION

Our study describes hepatic encephalopathy in a horse caused by *S.* *ovatus*. Many cases of *Senecio* spp. poisoning of horses have been described, but, as far as we are aware, there has not been any reported case of *S.* *ovatus* poisoning in any species. *S.* *jacobaea* is, by far, most frequently implicated as a source of PAs, but *S.* *brasiliensis, S.* *vulgaris*, *S.* *inaequidens* DC. and *S.* *erraticus* have been also reported (Gava and Barros 1997; Pohlmann et al. 2005; Passemard and Priymenko 2007; Panziera et al. 2017; Dau et al. 2019).

*S.* *ovatus* is a plant from the *Asteraceae* family with widespread distribution throughout Europe. It contains the PA senecionine, one of the most commonly encountered alkaloids in *Senecio* spp. (Mackay and Culvenor 1982; Roeder 2020). In our case, the mare must have eaten a significant amount of this plant repeatedly. The toxicity was due to a combination of relatively recent 24/7 access to the forest with *S.* *ovatus* without any supplementary forage. Due to her young age, probably she had not yet developed a tendency to avoid this plant.

Although it is described that horses usually avoid *Senecio* spp. on pastures due to the bitter taste, a study undertaken by Sroka et al. (2022) on hay showed that their rejection behaviour towards *S.* *jacobaea* differs individually. Individual variations in the susceptibility to the action of PAs have also been described (Giles 1983). According to the history of previous photodermatitis and alteration of liver biomarkers in horses from the same pasture, it is very likely that more horses developed a milder form of PA poisoning. Furthermore, on the same pasture, five days after the mare developed clinical signs, another mare was presented with a similar clinical picture and blood results: hyperbilirubinaemia (52 μmol/l, ref. range 13–34 μmol/l), elevated aspartate amino transferase (21.9 μkat/l, ref. range 1.7–5.83 μkat/l), gamma glutamyl transferase (4.79 μkat/l, ref. range 0.02–0.67 μkat/l) and alkaline phosphatase (17.87 μkat/l, ref. range 2.45–4.35 μkat/l). It seems that some horses avoided *S. ovatus* whereas others may have actively consumed it, which resulted in a hepatic disease of varying severity.

There are various causes of hepatic encephalopathy described in horses. In our case, PAs were mainly considered due to the presence of *S.* *ovatus* in the grazing area, which was eventually confirmed by determining their metabolites in the plasma and the liver. Other possible causes such as hepatic neoplasia, amyloidosis, liver parenchyma abscessation, fasciolosis and cholelithiasis were excluded from the post-mortem examination. Although certain mycotoxins may be present in the pasture, it is unlikely that such small amounts would induce hepatic encephalopathy. There were no fields or landfills in the vicinity, so poisoning by pesticides or heavy metals was also considered unlikely. Other causes of hepatic encephalopathy could be viral hepatitis caused by equine parvovirus and non-primate hepacivirus. Equine parvovirus hepatitis is associated with the recent administration of equine biological products and can be determined in the serum or liver by a real-time polymerase chain reaction (PCR) analysis (Divers 2014; Tomlinson et al. 2019). Because none of these products was administered in the herd, the probability of viral hepatitis was very low.

The mare developed bilateral laryngeal paralysis in connection with hepatic encephalopathy, which is considered a rare complication. The pathogenesis remains unclear, current studies point to the functional neuropathy of the larynx due to CNS damage (Johns et al. 2007). The blood tests revealed a concentration of bile acids higher than 50 μmol/l and a gamma glutamyl transferase activity higher than 6 μkat/l, both indicating a very poor prognosis (Mendel et al. 1988; Durham et al. 2003). It would be appropriate to determine the concentration of the conjugated bilirubin, which indicates biliary disease. Treatment of PA toxicosis consists of supportive therapy (Pohlmann et al. 2005). Diazepam was administered due to the seizures. Generally, benzodiazepines should be avoided in cases of hepatic encephalopathy, due to their ability to bind to gamma-aminobutyric acid (GABA) receptors and exacerbate any neuroinhibitory effects. At the time of admission, diazepam was administered because the whole case history was not known and the blood test results were not available. For sedation of patients with hepatic encephalopathy, pentobarbital, phenobarbital, detomidine or xylazine are the preferred choice (Weil 2011). Sodium bicarbonate was used to correct the metabolic acidosis. This is to be avoided in patients with liver failure, as a very fast correction of acidosis can increase the ammonia levels and contribute to further CNS damage (Divers 2014). It is recommended to administer fluids containing acetate such as Plasmalyte with 1–2.5% dextrose or Ringerfundine, which, unfortunately, were not available at our facility. Hypertonic saline (7.5% NaCl) was given to increase the intravascular fluid volume and alleviate any possible cerebral oedema. Furosemide was administered to prevent pulmonary oedema. For its anti-endotoxemic and antipyretic effects, flunixine was administered.

The results of the histopathological examination of the liver showed necrosis, fatty changes, haemorrhages and inflammation. Unlike our case, in most of the previous reports of *Senecio* spp. poisoning in horses (Gava and Barros 1997; Pohlmann et al. 2005; Dau et al. 2019), hepatic fibrosis, eventually cirrhosis, megalocytosis and biliary hyperplasia were seen.

Our case shows that the pathological finding may not always be specific. Acute toxicosis manifested by centrilobular liver necrosis with haemorrhage has also been described (Pohlmann et al. 2005). Probably the mare developed a subacute hepatic disease due to the ingestion of larger amounts of PAs in a shorter period, so the lesions characteristic of chronic PA poisoning could not be seen.

In our study, poisoning by PAs was confirmed by the determination of their metabolites in the liver and the plasma using the UHPLC-HRMS/MS method.
